# Vascular coil erosion into hepaticojejunostomy following hepatic arterial embolisation

**DOI:** 10.1186/s12893-015-0039-8

**Published:** 2015-04-29

**Authors:** Soondoos Raashed, Manju D Chandrasegaram, Khaled Alsaleh, Glen Schlaphoff, Neil D Merrett

**Affiliations:** Upper Gastrointestinal Unit, Bankstown Hospital, Sydney, Australia; Division of Surgery, School of Medicine, University of Western Sydney, Sydney, Australia; Interventional and Diagnostic Radiology, Liverpool Hospital, Sydney, Australia

## Abstract

**Background:**

Right hepatic arterial injury (RHAI) is the most common vascular injury sustained during laparoscopic cholecystectomy, occurring in up to 7% of cholecystectomies. RHAI is also the most common vascular injury associated with a bile duct injury (BDI) and is reported to occur in up to 41 – 61% of cases when routine angiography is employed following a BDI.

We present an unusual case of erosion of vascular coils from a previously embolised right hepatic artery into bilio-enteric anastomoses causing biliary obstruction. This is on a background of biliary reconstruction following a major BDI.

**Case presentation:**

A 37-year old man underwent a bile duct reconstruction following a major BDI (Strasberg-Bismuth E4 injury) sustained at laparoscopic cholecystectomy. He had two separate bilio-enteric anastomoses of the right and left hepatic ducts and had a modified Terblanche Roux-en-Y access limb formed.

Approximately three weeks later he was admitted for significant gastrointestinal bleeding and was hypotensive and anaemic. Selective computed tomography angiography revealed a 2 x 2 centimetre right hepatic artery pseudoaneurysm, which was urgently embolised with radiological coils.

Two months later he developed intermittent fevers, rigors, jaundice, and right upper quadrant pain with evidence of intrahepatic biliary dilatation on magnetic resonance cholangiopancreatography. The degree of intrahepatic biliary dilatation progressively increased on subsequent imaging over several months, suggesting stricturing of the bilio-enteric anastomoses. Several attempts to traverse these strictures with a percutaneous transhepatic approach had failed. Then, approximately ten months after the initial BDI repair, choledochoscopy through the Terblanche access limb revealed multiple radiological coils within the bilio-enteric anastomoses, which had eroded from the previously embolised right hepatic artery. A laparotomy was performed to remove the coils, take down the existing obstructed bilio-enteric anastomoses and revise this. Following this the patient recovered uneventfully.

**Conclusion:**

Obstructive jaundice and cholangitis secondary to erosion of angiographically placed embolisation coils is a rarely described complication. In view of the relative frequency of arterial injury and complications following major bile duct injury, we suggest that these patients be formally assessed for associated arterial injury following a major BDI.

## Background

Bile duct injury (BDI) associated with laparoscopic cholecystectomy (LC) occurs in 0.3 – 0.6% of cases [1]. Right hepatic arterial injury (RHAI) is the most common vascular injury during LC, occurring in up to 7% of cholecystectomies [2-4]. RHAI is also the commonest vascular injury associated with major BDI, with centers employing routine angiography following a BDI reporting RHAI rates of up to 41 – 61% [1,2,5], presumably secondary to the anatomical proximity of the right hepatic artery (RHA) to the bile duct (BD) [3,6]. Interestingly, where an associated biliary injury exists, arterial occlusions are far more common than pseudoaneurysms, however, where there is no biliary injury, reports of pseudoaneurysms are more common [3]. We present an unusual case of BDI repair complicated by RHA pseudoaneurysm requiring radiological coil embolisation, with a delayed complication of biliary obstruction secondary to erosion of coils into the hepato-enteric anastomosis.

## Case presentation

A previously healthy 37-year old man was transferred to our service with high volume bilious drainage immediately following an elective LC for gallstone pancreatitis. An endoscopic retrograde cholangiopancreatography (ERCP) showed no flow above the mid common bile duct (CBD). Laparotomy found that the CBD was divided at the level of the cystic duct with segmental resection of the common hepatic duct involving the confluence of the hepatic ducts with separation of the right and left hepatic ducts (Strasberg-Bismuth E4 injury). A bile duct reconstruction was performed, bringing together the separated adjacent walls of the right and left hepatic ducts together and suturing them to form a common wall. The bilio-enteric anastomoses were then performed over separate externalised stents via a modified Terblanche Roux-en-Y limb [7]. The anastomoses on subsequent imaging gave the appearance of a preserved confluence, because of the sutured common adjoining walls of the right and left separated ducts. There was an uneventful recovery and he was discharged 10 days post-operatively with the biliary stents in situ.

One week after discharge, the patient experienced a gastrointestinal bleed and presented with melaena to a nearby hospital. He was found to have a haemoglobin level of 90 g/L (Reference range: 130 – 180 g/L) and was transfused with two units of packed red blood cells. He underwent a gastroscopy and colonoscopy to investigate this. No obvious cause for his bleeding was found, and one colonic pedunculated polyp was removed. His bleeding settled and he was discharged three days after admission. Our unit was not notified of this admission.

**Figure 1:**
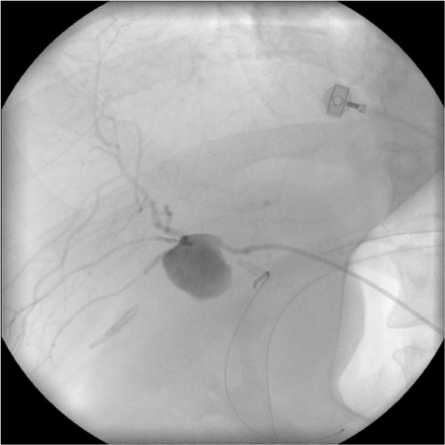
Pseudoaneurysm of the right hepatic artery on angiogram.

**Figure 2:**
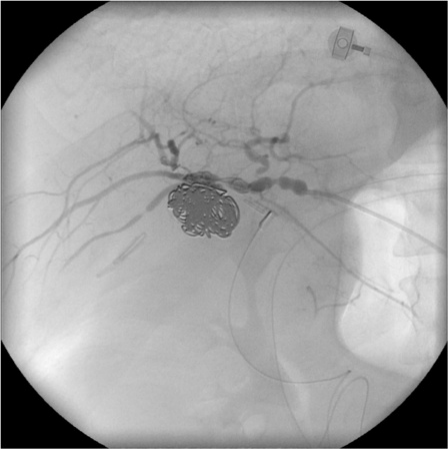
Coil embolisation of right hepatic arterial pseudoaneurysm.

Two days following this discharge, the patient had an episode of haematochezia associated with collapse at home. He was taken to the emergency department of our hospital where he was hypotensive (blood pressure 92/63 mmHg) and tachycardic (heart rate 115 beats/min). His abdomen was soft and non-tender, and digital rectal examination revealed dark red blood. His haemoglobin level was 111 g/L. He was initially resuscitated with crystalloids, but had further episodes of fresh rectal bleeding. Repeat haemoglobin was 58 g/L, and packed red blood cell transfusion was commenced. Urgent selective computed tomography angiography was performed revealing a 2 × 2 centimetre aneurysm arising from the RHA/cystic artery adjacent to surgical clips. The aneurysm was embolised with platinum and stainless steel radiological coils (Figure 1, Figure 2). A tubogram through the biliary stents after the procedure showed no evidence of leakage or stricture of the biliary anastomoses. He had no further bleeding and was discharged three days later. Four weeks later, the stents were removed in the outpatient rooms.

Two months later the patient developed intermittent fevers, rigors, jaundice and right upper quadrant pain. His liver function tests revealed a cholestatic picture with an elevated bilirubin of 59 μmol/L (Reference range: 0 – 17 μmol/L), ALP 473 U/L (Reference range: 30 – 115 U/L) and GGT 721 U/L (Reference range: < 66 U/L).

A magnetic resonance cholangiopancreatography (MRCP) was performed, showing slight prominence of the intrahepatic biliary radicles above the level of the right and left main hepatic ducts, with preservation of the contour of the biliary ducts without evidence of irregularity or stenosis. A hepatobiliary iminodiacetic acid (HIDA) scan to assess biliary excretion showed mild retention of tracer in left hepatic lobe inferiorly in pre- and post-cholecystokinin images but no evidence to suggest biliary stenosis. It also suggested poor emptying from the Roux loop and it was felt that associated bacterial overgrowth within the Roux loop may have been responsible for his symptoms and he was kept on low dose oral antibiotics with improvement of his liver function tests at six weeks (bilirubin 9 U/L, ALP 361 U/L and GGT 504). Progress MRCP two months later revealed increasing intrahepatic biliary dilatation with the left main hepatic duct increasing in diameter from 6.6 mm on the previous MRCP to 9.8 mm, suggesting a significant bilio-enteric anastomotic stricture.

**Figure 3:**
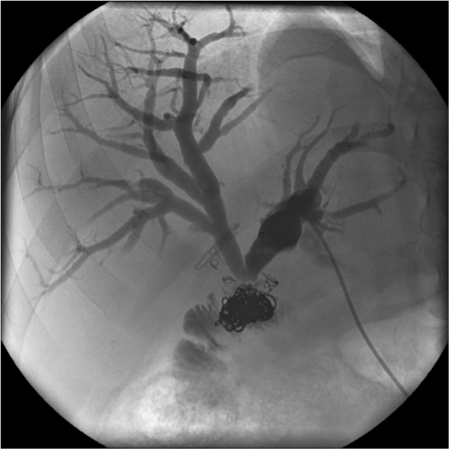
Biliary catheter in left hepatic ductal system.

**Figure 4:**
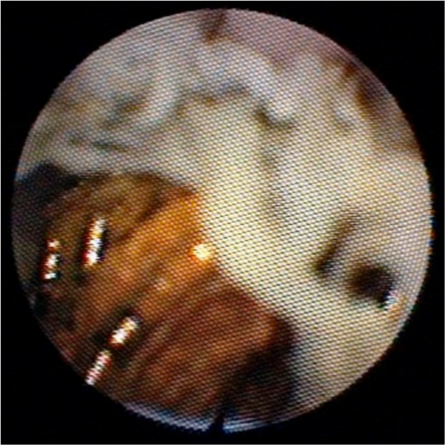
Coils seen around hepaticojejunostomy from within Roux limb on choledochoscopy.

**Figure 5:**
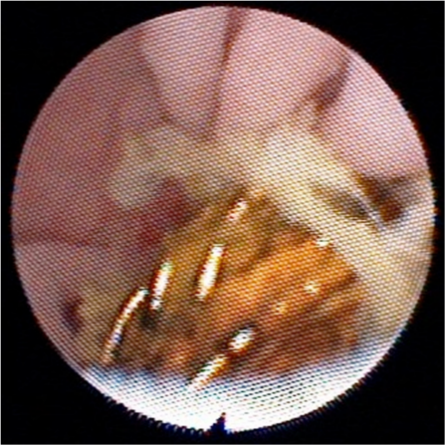
Coils seen around hepaticojejunostomy from within Roux limb on choledochoscopy.

Thus, approximately 10 months after his BD reconstruction, he was admitted electively for percutaneous transhepatic cholangiography (PTC) and balloon dilatation of this bilio-enteric anastomosis. After confirmation of the stricture by PTC, a 4 French catheter was inserted into his left ductal system and an 8.5 French pigtail catheter was placed into his right ductal system but neither could be advanced into the enteric limb (Figure 3). Two further attempts were made at PTC balloon dilatation, but both failed. It was decided to attempt to visualise the anastomosis through the Terblanche access limb. Choledochoscopy through the modified Terblanche access limb showed multiple radiological coils from the previous embolisation of the RHA pseudoaneurysm at the site of the anastomoses, causing a mechanical obstruction (Figure 4, Figure 5). Laparotomy was performed and the bilio-enteric anastomoses were taken down to remove the coils. The right and left ductal systems were evaluated with intraoperative cholangiogram and the bilio-enteric anastomoses were revised using the existing Roux limb around 10 French infant feeding catheters, which were externalised again through the modified Terblanche limb.

Post-operative recovery was uneventful. A cholangiography through the catheters showed good drainage of contrast through the bilio-enteric anastomoses. The patient was discharged 10 days after his operation, with total resolution of his jaundice and improvement in eating, drinking and general daily function. The stents were removed at six weeks and six years later the patient remained well with normal liver function tests and no further episodes of cholangitis.

## Discussion

From cadaveric studies, RHAI occurs in up to 7% of cholecystectomies. RHAI without concomitant BDI or portal vein injury rarely causes clinically significant liver or biliary ischaemia [2-4]. This may be due to the ability of the hilar marginal arteries to shunt blood from the left hepatic arterial system across to the remaining distal divided RHA system. It has been suggested that RHAI combined with biliary injuries can lead to ischaemic stricturing which may not become stable for several months following the injury and may be responsible for anastomotic stricturing after early repair [3]. Review of the vascularity of the BD has suggested that high anastomoses are to be preferred to low anastomoses [8]. Interestingly, there is disagreement as to whether a RHAI actually worsens biliary injury, which may relate to referral patterns and timing of repair [1]. Schmidt et al. found on univariate and mutivariate analyses that associated arterial injury and repair in the presence of active peritonitis were associated with increased complications after BDI [9]. However the delayed repair view is not universal, with many authors supporting early repairs [1,10,11]. A recent paper has emphasised that early repair by a specialist Hepatobiliary surgeon is associated with equivalent stricture rates to delayed repair but improved quality of life, return to normal activities and lower cost [12].

Hepatic artery pseudoaneurysm following LC has a reported incidence of 0.5 – 0.8% and can present four weeks post-operatively but may occur up to 13 months post-operatively [13-16]. Radiographic embolisation has been used successfully in the management of post-cholecystectomy pseudoaneurysms [13,15-18]. A variety of embolic agents have been described, including coils, Gelfoam, tissue adhesives, thrombin, detachable balloons and autologous clot [15]. Tulsyan et al. reported technical success in endovascular treatment of 11 hepatic artery pseudoaneurysms using coils, N-butylcyanoacrylate (N-BCA) glue or a combination of both depending on the desired rate of polymerisation [19]. The ideal placement of coils is a point of contention, with some authors advocating packing of the pseudoaneurysm itself and others suggesting “sandwich packing” (i.e. distal and proximal coil packing) to avoid rupturing the pseudoaneurysm [17], as well as to avoid coil migration [20,21]. In the elective setting, combination of stenting with a covered stent to maintain arterial flow and the packing and exclusion technique have been proposed, but no large series of this technique have yet been described and it may be problematic in the emergency setting [22-24].

Aneurysm coil migration from all sites into the gastrointestinal tract has been reported in at least twelve cases [25,26]. Coil migration into the CBD from the RHA has been reported in five cases (Table 1) [27,28]. Coil erosion into the BD or bilio-enteric anastomoses causing biliary obstruction is exceedingly rare, and we were only able to find three other reports of this in the literature [14,27,28].

**Table 1:** **Vascular coil migration from right hepatic artery to common bile duct**

**Author**	**Age/sex**	**Primary operation**	**Timing of RHApA bleed post-primary operation**	**Management of bleed**	**Time after which vascular coils migrated to CBD**	**Presenting symptom**	**Management**
Current study	38 M	Bile Duct Reconstruction after BDI following cholecystectomy	1 week	Required one attempt at coil embolisation “packing technique” with flow maintained within the artery.	10 months	Obstructive jaundice and Cholangitis	3 attempts with PTC to traverse biliary obstruction failed, bilateral biliary catheter drainage, and re-operation to revise hepaticojejunostomy
Van Steenbergen et al. [22]	72 M	Liver transplantation for primary biliary cirrhosis	10 weeks	Coil embolisation “packing technique” with flow maintained within the artery. Bleeding recurred with revascularization of aneurysm. ePTFE covered coronary stent placed to exclude pseudoaneurysm	5 years	Stone and coils in bile duct, described as “biliary colic”	ERCP (failed removal), coils and stone removed with PTC
AlGhamdi et al. [23]	55 F	Liver transplant for Hepatitis C cirrhosis and hepatocellular carcinoma	13 weeks post-transplant, (had 2 balloon angioplasties of hepatic artery jump graft 10 weeks post-transplant for stenosis)	Embolisation of bleeding aneurysm, and balloon covered stent used to treat hepatic artery stenosis. Further small pseudoaneurysm at junction of hepatic artery and jump graft managed with coil packing and further covered stent to exclude pseudoaneurysm.	3 months	Coil migration identified at time of biliary stent replacement for biliary stricture.	Coils and stones removed at ERCP with further balloon dilatation of stricture.
Turaga et al. [27]	65 M	Difficult cholecystectomy for gangrenous GB with T-tube choledochotomy after failed CBD stone retrieval	3 weeks	Required one attempt at embolisation	1 year	Obstructive jaundice and Cholangitis	ERCP (failed removal) ➔ required open bile duct exploration, removal of coils and insertion of T-tube. Artery and pseudoaneurysm ligated
Kao et al. [28]	65 F	Cholecystectomy and T-tube choledochostomy	Not reported	Coil embolisation	8 years	Obstructive jaundice	PTC performed for biliary drainage followed by ERCP for removal of coils and stone from CBD
Ozkan et al. [14]	58 M	Subtotal Cholecystectomy for cholecystitis	4 weeks, Required 2 attempts at embolisation	Coil embolisation, “packing technique” with flow maintained within the artery. Required further embolisation 3 days later for rebleed, and growth of neck of pseudoaneurysm	2 years	Pancreatitis	ERCP identified coils ➔ required open bile duct exploration, removal of coils and stones, and drainage of pseudocyst with cystojejunostomy

M: Male, F: Female, RHApA: RHA pseudoaneurysm, PTC: Percutaneous transhepatic cholangiography, ERCP: Endoscopic retrograde cholangiopancreatography.

## Conclusion

Obstructive jaundice and cholangitis secondary to erosion of angiographically placed embolisation coils is a rarely described complication following treatment of arterial injury subsequent to iatrogenic BDI. In view of the relative frequency of arterial injury and complications following major BDI, we would suggest that these patients be formally assessed for associated arterial injury.

## Consent

Written informed consent was obtained from the patient for publication of this Case report and any accompanying images. A copy of the written consent is available for review by the Editor of this journal.
